# Deranged Liver Function Tests and Liver Insults in Malnourished Patients: A Report of Two Cases and Literature Review

**DOI:** 10.7759/cureus.19607

**Published:** 2021-11-15

**Authors:** Joao Pinheiro, Ihab Jameel, Altaf Palejwala

**Affiliations:** 1 Gastroenterology and Hepatology, University Hospitals of Derby and Burton NHS Foundation Trust, Derby, GBR; 2 Gastroenterology and Hepatology, University Hospitals of Derby and Burton NHS Foundation Trust, Burton-On-Trent, GBR; 3 Gastroenterology, Queen’s Hospital Burton, Burton-On-Trent, GBR

**Keywords:** liver function, anorexia, hypertransaminaemia, malnourishment, hypoglycaemia, hepatocellular autophagy

## Abstract

Anorexia nervosa (AN) is an eating disorder often accompanied by complicated medical conditions. It often results in increased serum levels of liver enzymes, especially transaminases, and affects both males and females. Here, we describe the cases of two patients admitted to our District General Hospital. The patients presented with malnourishment secondary to AN and severely deranged liver function tests. According to our literature review, patients who are malnourished are particularly susceptible to liver injury, and small insults can amount to exaggerated liver inflammation with transaminitis. Once other aetiologies are excluded, this can be interpreted as a benign clinical event and is not associated with adverse events or higher mortality.

## Introduction

One of the most commonly occurring eating disorders is anorexia nervosa (AN) which usually begins in the teenage years. In AN, people experience disturbance in eating behaviour accompanied by a desire to lose weight. Individuals with AN may or may not have the habit of binge eating along with purging. These behaviours usually result in losing weight. Individuals suffering from AN have a fear of becoming fat and have a heightened desire to continue losing weight [1]. The lifetime prevalence of AN in females has been reported to be 1.2-2.2% according to large population-based studies, and the prevalence of the atypical type of AN has been reported to be 2.4 to 4.3% in females [2,3]. Smink et al. reported that the prevalence of AN in males is 10 times lower than in females [4]. Various chronic illnesses, co-morbidities, and high mortality rates are associated with AN among individuals suffering from psychiatric disorders [5]. Individuals with AN often present a challenge to doctors and therapists as they never accept that they are suffering from AN, or if they experience any signs and symptoms, they do not share them. Only 50% of AN cases get diagnosed [3], and out of these diagnosed cases, only one in three patients get specialist care [1].

AN is often accompanied by severe medical conditions such as haematologic disorders, electrolyte disorders, fractures, and bone density loss [6]. Gastric dilation, constipation, pancreatitis, and early satiety are well-known gastrointestinal manifestations associated with AN. An increase in liver enzymes is also common among individuals with AN. Injury to the liver cells can be detected by measuring serum aspartate aminotransferase (AST) and serum alanine aminotransferase (ALT). When hepatocytes are injured, the damaged cells release increased amounts of these enzymes into blood circulation. The mechanism involved in AN for the injury and death of liver cells is not completely known; however, it has been suggested that injury and cell death possibly occur due to starvation, which begins the process of autophagy. In this process, liver cells digest themselves to initially overcome any nutrient deficiency leading to cell death [7].

According to existing literature, liver enzyme levels and body weight have an inverse relation, suggesting that there is an increased risk of liver dysfunction with weight decreases in AN [8]. Moreover, it is known that inpatient refeeding in malnourished patients can also increase the levels of serum liver enzymes. A further complication associated with liver dysfunction and AN is hypoglycaemia [9]. Gibson et al. described that, in patients admitted with severe AN, 38% develop hypoglycaemia, with highly elevated liver function tests (LFTs) predicting its occurrence [10].

Here, we discuss our experience with two patients who were admitted with severe malnutrition secondary to AN with subsequent deranged LFTs.

## Case presentation

Case 1

A 45-year-old female was brought in by ambulance after collapsing at home secondary to a hypoglycemic event (capillary blood glucose of 1 mmol/L with paramedics). She had a history of restrictive AN, binge-purge behaviour, and an old traumatic brain injury, leaving her with memory problems. She was well known to mental health services, having been admitted multiple times to eating disorder centres for nasogastric feeding. She had never smoked in her life and denied any alcohol intake. The patient was on ferrous fumarate, fexofenadine, fluoxetine, ibuprofen, lansoprazole, quetiapine, supplemental vitamins, regular morphine (modified release), and gabapentin.

**Figure 1:**
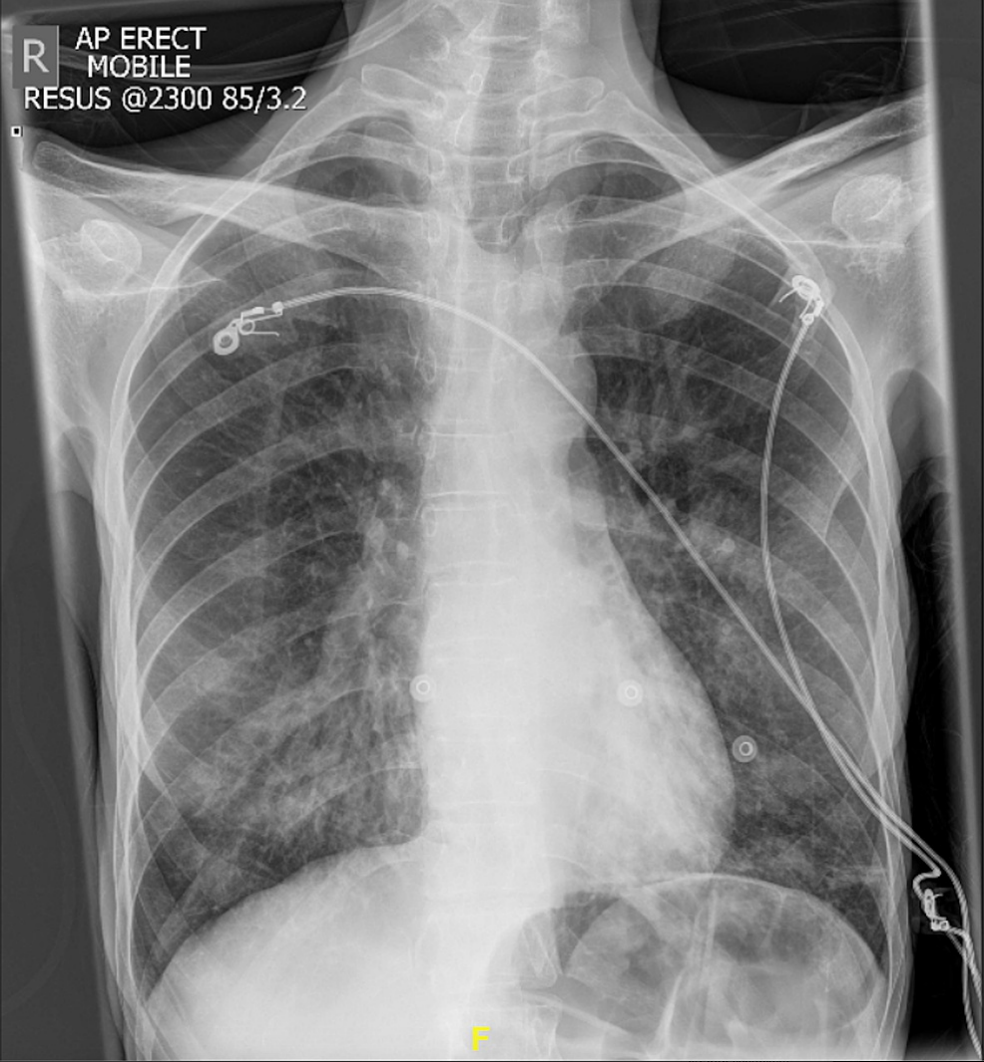
X-ray of the chest at the time of admission showing opacification in the right middle and lower zones.

On admission, her blood pressure was 106/85 mmHg, respiratory rate was 20 breaths/minute, heart rate was 64 beats/minute, temperature was 35.1 °C, and capillary blood glucose was 6 mmol/L. Her weight on admission was 37.3 kg (body mass index [BMI] = 12.6). On examination, she was clearly malnourished, cachexic, and dehydrated. The rest of the clinical examination was normal, as shown in Table 1. Her chest radiograph showed patchy consolidations in the right middle and lower lobes (Figure 1). She was prescribed appropriate antibiotics. She was refusing treatment and was deemed to lack the capacity to make that decision. Therefore, Section 5(2) under the Mental Health Act was put in place. She was commenced on oral supplements as per guidance from the dietitian, and then switched to nasogastric feeding.

**Table 1:** Blood results at the time of admission. WBC: white blood cell; Hbg: haemoglobin; ALT: alanine aminotransferase; AST: aspartate transaminase; CRP: C-reactive protein

	Results	Units	Normal range
WBC	4.6	10^9^/L	4.0–10.0
Hbg	134	g/L	120–150
Sodium	134	mmol/L	133–146
Potassium	5.3	mmol/L	3.5–5.3
Urea	22.9	mmol/L	2.7–7.8
Creatinine	116	µmol/L	44–80
Bilirubin	8	µmol/L	0.0–21
ALT	385	U/L	0.0–33
ALP	675	U/L	30–130
Total protein	68	g/L	60–80
CRP	24	g/L	18–35

**Figure 2:**
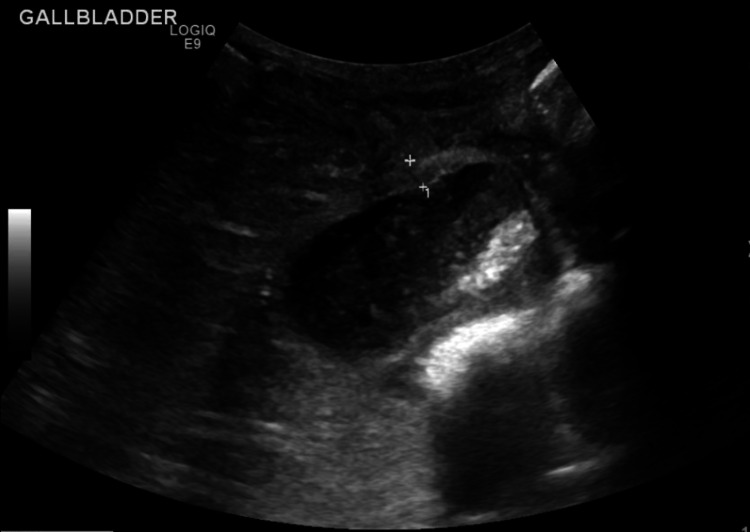
Ultrasound showing biliary sludge and some peri-cholecystic fluid.

On the night of the second day, she had an episode of decreased consciousness, bradypnoea (RR-6), and hypotension (83/64). Her blood sugar level was 6.6 mmol/L. After receiving Naloxone, her symptoms improved, and her opiates were discontinued. The following day she mentioned right upper quadrant pain. Blood tests showed worsening liver enzymes, as shown in Table 2. An abdominal ultrasound was performed on the fourth day of admission, which showed multiple small calculi and biliary debris, with gallbladder oedema, and a small amount of peri-cholecystic fluid (Figure 2).

**Table 2:** Evolution of liver function tests. ALT: alanine aminotransferase; AST: aspartate transaminase

Test	Fourth day	Sixth day	Fourth week	Unit	Normal range
Bilirubin	20	25	4	µmol/L	0.0–21
ALT	1,268	1,481	35	U/L	0.0–33
ALP	1,945	1,344	295	U/L	30–130
Total protein	48	57	6	g/L	60–80
Albumin	24	16	35	g/L	35–50

She was treated for cholecystitis with amoxicillin, clavulanic acid, and clarithromycin, which led to improvement in her inflammatory markers in the following days. She underwent an inpatient magnetic resonance cholangiopancreatography (MRCP) to rule out intra-biliary pathology, which was negative. Subsequently, she was referred to surgeons for consideration of elective laparoscopic cholecystectomy once deemed fit.

She had a protracted and difficult admission. Due to the coronavirus disease 2019 pandemic, her transfer to an Eating Disorders Unit proved difficult. By the fourth week of admission, her liver enzymes had improved, as can be seen in Table 2. Nasogastric feeding was successfully weaned as her weight improved. She was discharged on day 51 to an Eating Disorders Unit weighing 56.4 kg (BMI = 18.1) from her admission weight of 37.3 kg (BMI = 12.6).

Case 2

A 29-year-old male was brought to the hospital by ambulance after collapsing at home. He was found to be bradycardic and hypoglycaemic with a capillary blood glucose level of 2.3 mmol/L. He had a history of eating and anxiety disorders and was not on any regular medications. On admission, his weight was 37.3 kg (BMI = 11.6). His blood pressure was initially un-recordable but subsequently was recorded to be 104/72 mmHg. His capillary blood glucose level was 4.7 mmol/L, and his Glasgow Coma Scale score was 15/15. On examination, he was noted to be severely malnourished and cachexic. The rest of the clinical examination was normal. LFTs were very abnormal, as shown in Table 3.

**Table 3:** Blood results at the time of admission. WBC: white blood cell; Hbg: haemoglobin; MCV: mean corpuscular volume; ALT: alanine aminotransferase; AST: aspartate transaminase; INR: international normalised ratio

	Results	Units	Normal range
WBC	152	g/L	4.0–10.0
Hbg	152	g/L	120–150
MCV	88	fL	83–101
Platelets	104	10^9^/L	150–410
Sodium	131	mmol/L	133–146
Potassium	3.9	mmol/L	3.5–5.3
Bilirubin	55	µmol/L	0.0–21
ALT	997	U/L	0.0–33
ALP	134	U/L	30–130
Albumin	42	g/L	35–50
INR	1.9		0.8–1.2

Since admission, he seemed to lack insight. Due to problems keeping him compliant with medication and intravenous glucose, he had a number of hypoglycaemic events in the first two days of admission. The following day, he was deemed not to have the capacity. He underwent Mental Capacity Assessment and Deprivation of Liberty Safeguards. He was ultimately placed under Mental Health Act 5(2) and was started on nasogastric feeding. His liver enzymes worsened further after the introduction of nasogastric feeding, but we were reassured by a normal non-invasive liver screen and ultrasound.

His condition, liver tests, and liver synthetic function improved over the course of his 24-day admission (Table 4), but his stay was associated with difficult behaviour. He was eventually discharged to an Eating Disorders Unit.

**Table 4:** Evolution of the patient’s liver function tests. ALT: alanine aminotransferase; AST: aspartate transaminase; INR: international normalised ratio

	First day	Sixth day	Ninth day	Nineteenth day	Unit	Normal range
Bilirubin	55	111	105	32	µmol/L	0.0–21
ALT	997	1,634	919	132	U/L	0.0–33
ALP	134	454	339	204	U/L	30–130
Total protein	NA	55	NA	55	g/L	60–80
Albumin	42	38	33	33	g/L	35–50
INR	1.9		1.1			0.8–1.2

## Discussion

AN is a very serious and potentially life-threatening condition and is associated with multiple co-morbidities. Derangement of LFTs has been described in the literature, with mild derangement being the most common in the outpatient setting [6] and acute liver injury in the inpatient setting [8,11,12]. The pathophysiologic mechanisms for hypertransaminasemia in malnourished individuals include non-alcoholic fatty liver disease, as well as hypoperfusion, hypothermia, hepatotoxic medications, and hepatotropic viruses. Hepatocellular autophagy in malnourishment has been described by Restellini et al. [12]. Even though the pathophysiology remains unclear, it is clear that malnutrition lowers the threshold for significant liver injury, regardless of the causative process. This is due to a lack of glycogen stores and reduced liver function reservoir. Hanachi et al. [13] have described four risk factors for hypertransaminasemia in malnourished individuals with AN, namely, young age, low BMI, male sex, and the pure restrictive form of the disease.

Resolution of transaminitis occurs as BMI improves [5,6]. Although increased levels of transaminases are seen in AN [7,9], levels more than 10 times the normal are unusual. This is more common in patients with a BMI of less than 12 kg/m^2^ [11]. It can be suggested that the starvation state may be an inherent risk in AN because of the inverse relationship of BMI with transaminase levels [11]. Although refeeding transaminitis is well documented, both of our cases had an abnormal rise in enzymes before any feeding was instigated. In both of our cases, we assume hypoglycaemia was the main trigger of transaminitis/acute hepatitis [14,15].

We observed an increase in liver enzyme levels in the first few days of hospitalisation. This shows that the likely reason for the liver injury was starvation rather then steatosis due to refeeding. Case 1 was complicated as there was an underlying chest infection and cholecystitis which may have added to the elevated liver enzyme levels. However, the degree of elevation seems to have been greatly exaggerated. Case 2 appears to have had a more direct correlation with hypoglycaemia causing liver insult. His other risk factors could be hypotension-induced liver injury, but this would be unusual in a young man without a history of liver disease and comorbidity. In both cases, autophagy-related hepatocellular injury may have been the underlying cause.

## Conclusions

This report adds to the existing literature related to the elevation of transaminases in AN and malnutrition. Transaminitis in this context, once other causes are excluded, should be considered as an exaggerated liver insult in a patient with poor functional physiological reserve. We conclude that deranged liver enzymes in this cohort does not constitute a predictor for adverse liver outcomes but reflects poor liver reserve secondary to malnutrition. The exact pathogenesis as to why this occurs remains unclear.
